# Fluoride‐Free 2D Niobium Carbide MXenes as Stable and Biocompatible Nanoplatforms for Electrochemical Biosensors with Ultrahigh Sensitivity

**DOI:** 10.1002/advs.202001546

**Published:** 2020-11-09

**Authors:** Menglin Song, Sin‐Yi Pang, Feng Guo, Man‐Chung Wong, Jianhua Hao

**Affiliations:** ^1^ Department of Applied Physics The Hong Kong Polytechnic University Hong Kong 999077 P. R. China

**Keywords:** 2D MXene, electrochemical exfoliation, enzyme biosensors, fluoride‐free Nb_2_CT*_x_*, phosmet detection

## Abstract

Recently, 2D niobium carbide MXene has drawn vast attention due to its merits of large surface area, good metallic conductivity, and tunable band gap, making it desirable for various applications. However, the usage of highly toxic fluoride‐containing etchant and quite long etching time in the conventional synthesis route has greatly hindered further exploration of MXene, especially restricting its biomedical application. Herein, novel fluoride‐free Nb_2_CT*_x_* nanosheets are prepared by a facile strategy of electrochemical etching (E‐etching) exfoliation. Taking advantage of rapid aluminum clearance, excellent chemical stability, and biocompatibility from the MXene by E‐etching, fluoride‐free Nb_2_CT*_x_*/acetylcholinesterase‐based biosensors are constructed for phosmet detection with the limit of detection down to 0.046 ng mL^−1^. The fabricated Nb_2_CT*_x_*‐based biosensor is superior to the counterpart from hydrofluoric acid‐etched Nb_2_CT*_x_*, indicating that fluoride‐free MXene can enhance the enzyme activity and electron transfer in the biosensor. The results prove that the fluorine‐free MXene shows promise for developing biosensors with high performance of ultrahigh sensitivity and selectivity. It is highly expected that the fluoride‐free MXene as a stable and biocompatible nanoplatform has great potential to be expanded to many other biomedical fields.

## Graphical abstract

Fluoride‐free Nb_2_CT_*x*_ nanosheets are prepared by a facile strategy of electrochemical etching and are engineered into an enzymebased nanoplatform to achieve a detection limit of 0.046 ng mL^−1^ for phosmet detection, outperforming the counterpart of hydrofluoric acid (HF) etched Nb_2_CT_*x*_. The results prove that the fluorine‐free MXene can be used in biosensors with ultrahigh sensitivity and selectivity.

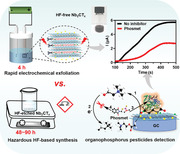

MXenes as an emerging class of 2D materials are composed of layered transition metal carbides or nitrides with high conductivity, good hydrophilicity, superior optical, and mechanical properties.^[^ ^1^ ^]^ MXenes have been developed in various applications, such as electrochemical catalysis,^[^ ^2^ ^]^ medical devices,^[^ ^3^ ^]^ energy conversion, and storage.^[^ ^4^ ^]^ MXenes can be generally classified as M*_n_*_+1_X*_n_*T*_x_* (*n* = 1, 2, or 3) where *n* + 1 layers of transition metals M (e.g., Sc, Ti, V, and Nb) are interleaved with *n* layers of X (C and/or N) and T*_x_* represents various surface terminations, such as —OH, —O, —F, and —Cl.^[^ ^5^ ^]^ Typically, MXenes are synthesized by selectively etching the “A” layers from MAX (A is mainly a group IIIA or IV element, such as Al) phased powders with excess hydrofluoric acid (HF) or in situ formation of HF species. The selective etching process is achieved by employing the difference in M—X and M—A bonding since metallic M—A bonds are usually weaker than M—X.^[^ ^6^ ^]^ So far, more than 20 different types of MXenes have been experimentally obtained and over 70 MXenes have been theoretically predicted.^[^ ^7^ ^]^ Among these different composites of ordered double MXenes, niobium carbide (Nb_2_CT*_x_*) possesses its specificity in metallic properties through a flexible combination of functional groups.^[^ ^8^ ^]^ Nb_2_CT*_x_* holds excellent conductivity with almost zero band gap,^[^ ^9^ ^]^ coupled with hydrophilic nature, and other unique physiochemical performances, distinguishing it from other traditional 2D materials. Nb_2_CT*_x_* has been investigated as electrodes for Li‐ion energy storage devices,^[^ ^10^ ^]^ dye adsorption,^[^ ^11^ ^]^ and fluorescence imaging.^[^ ^12^ ^]^ However, there are very few investigations on Nb_2_CT*_x_* regarding biomedical applications, especially biosensing applications.^[^ ^13^ ^]^ Underlying reasons of such obstructions can be summarized as follows: i) the synthesis route of Nb_2_CT*_x_* is time consuming and it has a high risk of exposure to corrosive HF acid, usually requires a relatively long reaction time (48–90 h) and highly concentrated (50% conc.) HF solution not only makes the preparation of MXenes more hazardous but also increases the complexity of the processes.^[^ ^10^, ^13^, ^14^ ^]^ ii) Although HF or in situ HF etching method are potent, complete removal of A element (Al) sometimes could not be achieved by this approach according to some previous reports.^[^ ^11^ ^]^ iii) Due to excessive use of HF acid, the F^−^ residues on the surface of Nb_2_CT*_x_* are prone to release HF during the hydrolysis reactions or electrochemical reactions.^[^ ^15^ ^]^ The potential toxicity of HF would relatively reduce the enzyme activity or cause a significant damage to the normal cells in vivo, which dramatically hinders the further exploration of Nb_2_CT*_x_* in biomedical applications.^[^ ^16^ ^]^ iv) In view of the flexible terminations of HF‐etched Nb_2_CT*_x_*, it is easy to be oxidized under ambient environment or during the oxygen evolution reaction (OER) process,^[^ ^17^ ^]^ severely affecting the instability and effectiveness of Nb_2_CT*_x_* in biosensing process. Recently, Yang et al. reported that MXene could be obtained by the E‐etching method of tailoring the electrolyte composition (ammonium chloride + tetramethylammonium hydroxide).^[^ ^18^ ^]^ However, the alkaline nature of tetramethylammonium hydroxide led to the unfavorable formation of MXene quantum dots.^[^ ^19^ ^]^ Furthermore, the previously reported E‐etching method is limited to investigating titanium carbide (Ti_2_C or Ti_3_C_2_). Due to the difference in the bond strength of M—A bonding of their parent MAX phase materials and the potentially impractical etching in the relatively mild environment, it is necessary to further verify whether other types of MXene can be obtained by E‐etching. Therefore, from the perspective of biomedical systems, a safe, robust, and fluorine‐free synthesis method for producing stable and biocompatible Nb_2_CT*_x_* MXene nanosheet is essentially required.

On the other hand, organophosphorus pesticides (OPs) have been substantially utilized to eliminate the insect in agricultural industry to increase crop productivity. Although these toxic compounds contribute to increasing agricultural productivity, the overuse of OPs can lead to negative effects on animals and humans. These OPs, even used in very small amounts, may accumulate in the human body through ecosystem cycles, which allows rapid inhibition of the activity of acetylcholinesterase and subsequently prevent the transmission of neurotransmitters, resulting in nervous system dysfunction and physical discomfort and even death.^[^ ^20^ ^]^ Recently, some strategies including high‐performance liquid chromatography, gas chromatography (GC), mass spectrometry, enzyme linked immunosorbent assay, have been employed to quantify OPs.^[^ ^21^ ^]^ Although these methods have shown good sensitivity and high selectivity for OPs detection, they still suffer from some serious limitations, such as expensive equipment, complicated procedure, long‐time analysis process, and limited on‐site and real‐time detection, which significantly hamper their further development.^[^ ^22^ ^]^ As such, low cytotoxicity, high porosity, and excellent conductivity of aforementioned fluorine‐free Nb_2_CT*_x_* are well suitable for the enzymatic biosensor, especially, the unique metallicity and large surface area of the fluorine‐free Nb_2_CT*_x_* are beneficial to enhance the response signal of the biosensing system and realize accurate on‐site detection and quantification of OPs in environment and biological samples.

Here, we synthesized 2D fluoride‐free Nb_2_CT*_x_* MXene nanosheets through an electrochemical etching (E‐etching) exfoliation route developed from our recently proposed strategy for HF‐free synthesis of MXenes used in energy applications.^[^ ^23^ ^]^ Taking advantage of endowments in low cytotoxicity, good chemical stability, high porosity, and excellent conductivity of fluorine‐free Nb_2_CT*_x_*, we here fabricated an enzymatic electrochemical biosensor for ultrasensitive pesticide phosmet detection (**Figure** **1**). Compared with HF‐etched counterpart, the fluorine‐free Nb_2_CT*_x_* based biosensor shows good stability and biocompatibility, resulting from stabilized enzyme activity and enhanced electrochemical activity, indicating the presented biosensor possesses ultrahigh sensitivity and capability of on‐site and real‐time phosmet detection. Through the enzyme inhibition effect, the robust platform can realize a limit of detection (LOD) for sensing phosmet down to 144 × 10^−12^ m (0.046 ng mL^−1^), which is much lower than the required residue limit (10 ppb) set by the U.S. Environmental Protection Agency.

**Figure 1:**
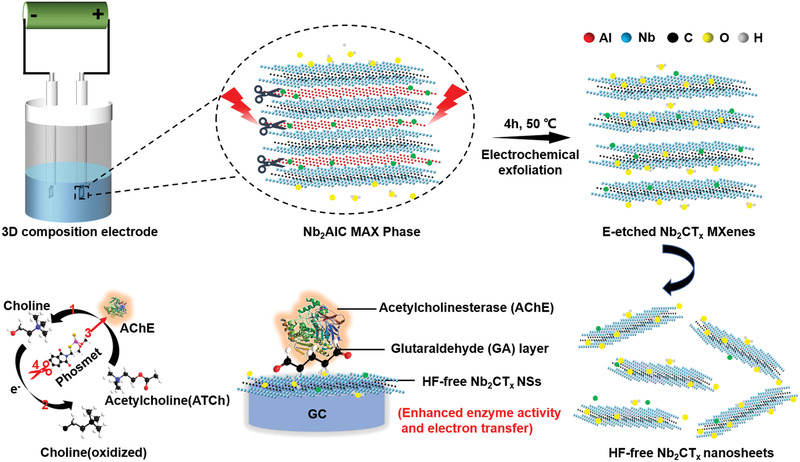
Schematic for exfoliation and delamination process of Nb_2_AlC MAX phase via electrochemical etching and the enzyme inhibition effect for phosmet detection by HF‐free Nb_2_CT_*x*_/AChE based biosensor.

Achieving electrochemical exfoliation of pristine bulk MAX phase material to a few layers was verified by transmission/scanning electron microscopy (TEM/SEM), high‐resolution TEM (HRTEM) images, and Raman spectroscopy. The rapid and facile exfoliation and delamination process of Nb_2_AlC MAX phase are achieved by a strategy of electrochemical etching. SEM images show the Nb_2_AlC MAX phase before exfoliation (**Figure** **2**a). As demonstrated in Figure 2b, the exfoliated Nb_2_CT_*x*_ of MXene by E‐etching is sheet‐like and near to transparency, indicating its ultrathin thickness. HRTEM image in Figure 2c manifests the crystalline lattice of few‐layer or single‐layer Nb_2_CT_*x*_ nanosheets with hexagonal structure that was further confirmed through selected area electron diffraction (SAED) pattern (Figure 2d). The hydrodynamic diameter of the as‐synthesized E‐etched Nb_2_CT_*x*_ aqueous solution was calculated to be 360 ± 2.3 nm by dynamic light scattering (DLS) analysis and underwent a significant downsizing from the bulk (average size: 933.47 nm) (Figure 2e). As shown in Figure 2f, the zeta potential *ζ* of the Nb_2_AlC was measured to be +24.0 mV, and the *ζ* of E‐etched Nb_2_CT_*x*_ nanosheet was changed to be −26.2 mV, which is primarily ascribed to the presence of flexible terminations, evidencing successful exfoliation by the E‐etching method. UV–vis–near‐infrared (NIR) spectra of E‐etched Nb_2_CT_*x*_ show strong photo‐absorption in the NIR range as well as in the second bio‐window (Figure 2g). The measured result indicates that Nb_2_CT_*x*_ has highly photothermal conversion potential in both NIR‐I and NIR‐II bio‐windows, which should be beneficial for the future expanded biological NIR related applications.^[^ ^24^ ^]^ As shown in Figure 2h, the Raman spectra of Nb_2_AlC and Nb_2_CT_*x*_ nanosheets illustrate distinct vibration peaks redshift from 127 to 180 and 260–270 cm^−1^ and the vibration peaks at 850 and 980 cm^−1^ became suppressed or even vanished after E‐etching, implying the elimination of Nb‐Al bond or the exchange‐out of Al atoms by lighter atoms. Importantly, owing to its rich hydroxyl group on the surface, the prepared Nb_2_CT_*x*_ can be evenly dispersed in the water phase (Figure 2i), which brings various advantages to the application of enzyme‐based biosensors, including great convenience, enhanced biosensors stability, and reproducibility.

**Figure 2:**
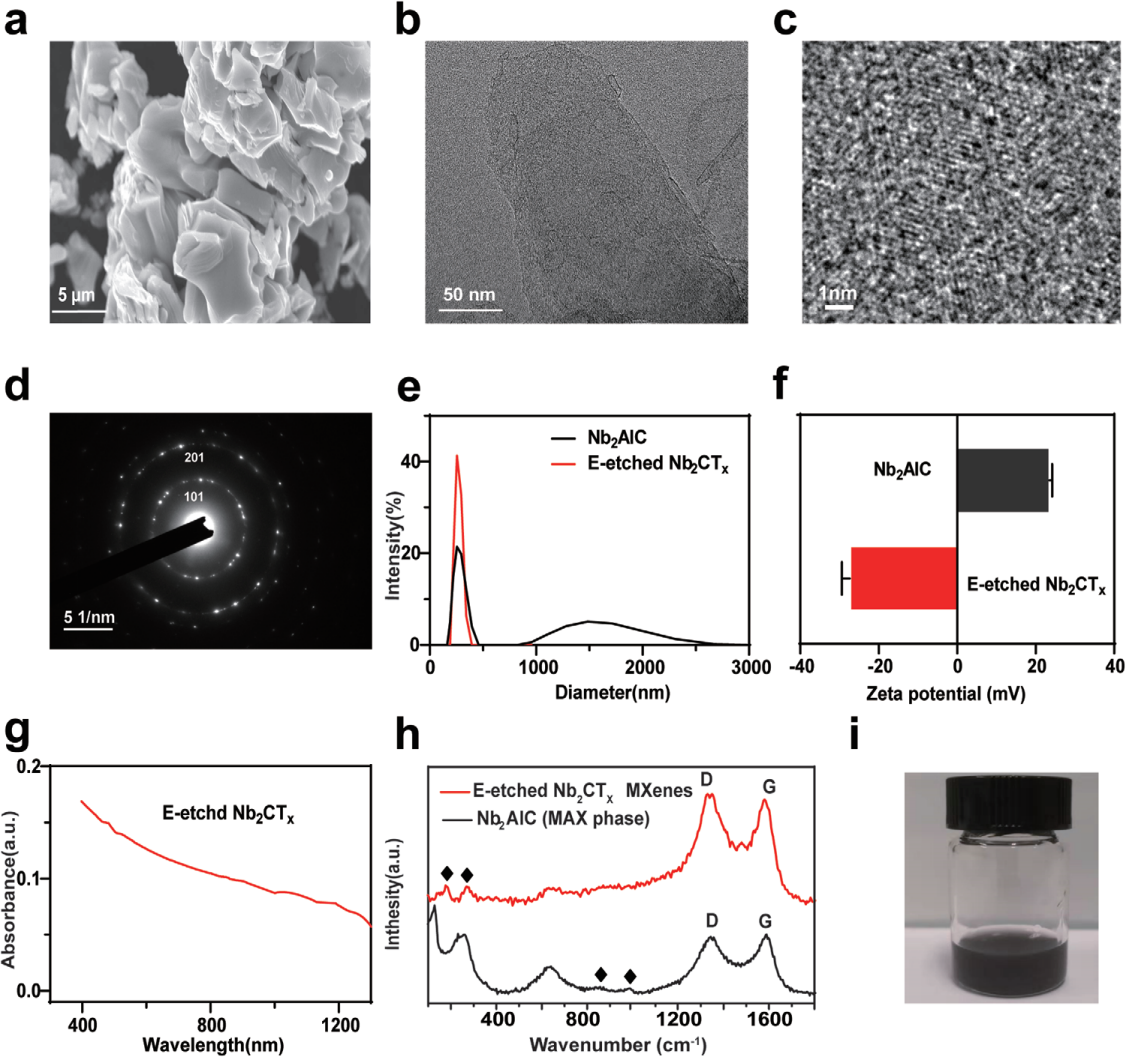
a) SEM image of Nb_2_AlC MAX phase. b) TEM image of E‐etching preparation of Nb_2_CT_*x*_nanosheets, c) HRTEM image, and d) SAED pattern ofE‐etched Nb_2_CT_*x*_. e) Dynamic light scattering (DLS) size distribution profiles and f) Zeta potential of E‐etched Nb_2_CT_*x*_ and Nb_2_AlC dispersed in aqueous solution. g) UV–vis–NIR spectra of E‐etched Nb_2_CT_*x*_nanosheets. h) The Raman spectra of Nb_2_AlC and E‐etched nanosheets. i) The photography of the E‐etched Nb_2_CT_*x*_nanosheets dispersion.

It has been well documented that the Nb_2_CT_*x*_ can be etched by the conventional corrosive HF acid solution within 48–90 h (Table S1, Supporting Information). Although HF or in situ HF etching method is extensively used for MXenes production, the synthesis process requires relatively long etching time and extra protective procedures. In addition to the fluorine residues on the surface after synthesis, HF‐etched Nb_2_CT_*x*_ is also prone to be oxidized under ambient environment or during the OER process,^[^ ^16^ ^]^ significantly affecting the instability and effectiveness of Nb_2_CT_*x*_ nanosheets in biomedical or other applications. The structure and phase of the Nb_2_CT_*x*_ nanosheets were further examined by X‐ray diffraction (XRD) and X‐ray photoelectron spectroscopy (XPS). The MXene was scanned over the 2*θ* range of 5°–60° to identify the crystal structure. As shown in **Figure** **3**a,b, the (002) peak of Nb_2_CT_*x*_ nanosheets exfoliated by two etching methods both broadened and downshifted in the XRD patterns toward a lower 2*θ* angle of 7.39° and 5.86°, respectively. Such observations are in good agreement with those previously reported literatures,^[^ ^13^, ^14^ ^]^ indicating the etched Nb_2_CT_*x*_ is crystallized. Nevertheless, some peaks in 46.4° and 56.3° are observed in the XRD pattern of HF‐etched Nb_2_CT_*x*_ and this is indicative of the presence of orthorhombic Nb_2_O_5_ in the oxidized HF‐etched Nb_2_CT_*x*_ samples, which is consistent with the previous report on the oxidation of Nb_2_CT_*x*_.^[^ ^25^ ^]^ TEM images of the HF‐etched Nb_2_CT_*x*_ in Figure 3c and Figure S2 in the Supporting Information are in line with the results in which some small‐sized nanoparticles of NbO_*x*_ were formed on the surface of the MXene. Compared with the conventional HF etching method, the E‐etched method offers the higher degree of Al layer removal (Figure 3d) and high‐resolution XPS survey of E‐etched Nb_2_CT_*x*_ on Al 2p demonstrates the vanished Al binding energy signal on 75 eV (Figure 3e). It suggests the success of selective E‐etching of Al from its MAX phase, although there exists a small peak on Al for the HF‐etched Nb_2_CT_*x*_ MXene. Figure 3f manifests high‐resolution XPS on F 1s and unveils the presence of F functionalized Nb_2_CT_*x*_ bonding. The electron energy loss spectrum of HF‐etched Nb_2_CT_*x*_ nanosheets (Figure S3, Supporting Information) also shows typical F and O signal, revealing the residue of F and the potential toxicity for biosystems after the HF etching and which is consistent with the XPS result.

**Figure 3:**
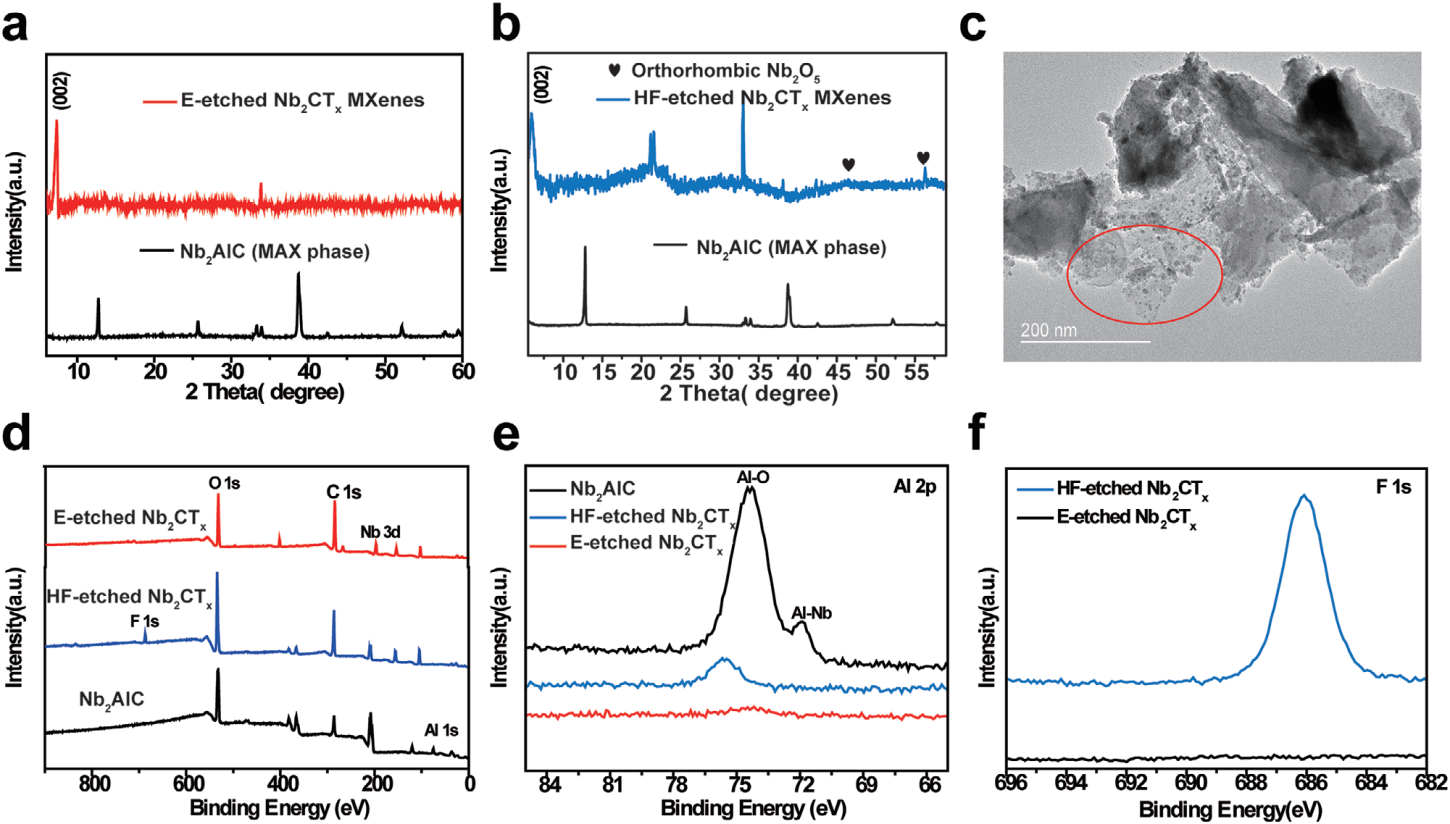
XRD patterns of a) E‐etched Nb_2_CT_*x*_nanosheets and b) HF‐etched Nb_2_CT_*x*_nanosheets. c) Transmission electron microscopy image of HF‐etched preparation of Nb_2_CT_*x*_MXene, the black dots in red circle indicate NbO_*x*_nanoparticles formed by oxidation. d) XPS survey and high‐resolution deconvoluted spectrum of HF‐etched Nb_2_CT_*x*_and E‐etched Nb_2_CT_*x*_on e) Al 2p and f) F 1s.

Benefiting from 2D morphology with large surface area with 35.3 m^2^ g^−1^ (Figure S4, Supporting Information), anisotropic electron transport behavior, outstanding electroconductivity as well as excellent biocompatibility of the E‐etched Nb_2_CT_*x*_, we then constructed the fluorine‐free Nb_2_CT_*x*_/acetylcholinesterase (AChE) based biosystem for OPs detection by an enzyme inhibition effect. The inhibition effect of phosmet on HF‐free Nb_2_CT_*x*_/AChE based biosensor is illustrated in **Figure** **4**a and the biosensing system presents high electrochemical reversibility and stability after a prolonged period (Figure S6, Supporting Information). The proposed biosensor was fabricated with composites in which glutaraldehyde (GA) layer was coated on a glassy carbon electrode (GCE) before immobilization with AChE that can catalyze neurotransmitter acetylcholine into choline and acetic acid. Due to electroactivity of choline, it can be easily oxidized and detected in an electrochemical way.^[^ ^26^ ^]^ Organophosphate pesticide phosmet is an irreversible inhibitor that can form a strong covalent bond between the phosphate group of pesticide and the hydroxyl group in the active site of the enzyme, which results in the formation of a stable enzyme‐pesticide complex and inhibition of enzyme activity. By the inhibition effect, the percentage of inhibition can be calculated based on different concentration of phosmet. Subsequently, we evaluated different E‐etched MXenes (Nb_2_CT_*x*_, V_2_CT_*x*_, Ti_3_C_2_T_*x*_) biosensor systems with successive injection of acetylcholine (ATCh) to verify the feasibility of the phosmet detection. As shown in Figure 4b, biosensors based on these three materials exhibit much higher performance compared to the biosensors without MXene, which is attributed to the enhanced enzyme activity and electron transfer ability of HF‐free MXenes based platform (Figure S7, Supporting Information). Among various MXene‐based biosensors, Nb_2_CT_*x*_ based biosensor system also outperforms the counterpart of V_2_CT_*x*_ and Ti_3_C_2_T_*x*_ due to its excellent metallic properties with near‐zero energy bandgap.^[^ ^9^ ^]^ In sharp contrast to Nb_2_CT_*x*_, other reported ordered double MXenes are semiconducting when they are functionalized by —O/—OH/—F, exhibiting a lower conductivity when compared to the metallic material. Furthermore, the lower contact resistance of Nb_2_CT_*x*_ is shown in Figure S8 in the Supporting Information, illustrating the superior high conductivity of the Nb_2_CT_*x*_ when compared to Ti/V‐based MXene in practical application. All these results reveal that E‐etched Nb_2_CT_*x*_ based biosensor is a promising candidate for the phosmet sensing. And then we used chronoamperometry to observe the chronoamperometric response signal after injection of successive ATCh into different modified layers. As can be seen from Figure 4c, there is no obvious current response when measuring on individual glassy carbon. When GC is modified with E‐etched Nb_2_CT_*x*_, the current response will be increased slightly after ATCh injection (0.39 µA). The signal generated may come from the oxidation of a small amount of iodine used with ATCh.^[^ ^27^ ^]^ Besides, GC/E‐etched Nb_2_CT_*x*_/GA shows a low current response (0.41 µA), manifesting the absence of catalytic activity and important functionalities of AChE. After immobilization of AChE, the response signal increases dramatically (3.72 µA), which can be attributed to the enzyme's high turnover and powerful catalytic ability.^[^ ^28^ ^]^ Stronger current response was measured with an increment of ATCh concentration. By the action of acetylcholinesterase, more amount of choline will be produced and subsequently oxidized to generate an intense current signal. Figure 4d also demonstrates the significance of the enzyme and there is no oxidation peak on the anode without AChE coating. It is noted that the F^−^ residues on the surface of HF‐etched Nb_2_CT_*x*_ are prone to release HF during the hydrolysis reactions or hydrogen evolution reaction process and the released HF has an adverse effect on enzyme, thereby reducing the performance of biosensor. The absence of the irreversible oxidation peak at +270 mV as shown in Figure 4e and Figure S10 in the Supporting Information implies an enhanced electrochemical stability exhibited by our GCE/E‐etched‐Nb_2_CT_*x*_ as compared with the GCE/HF‐etched Nb_2_CT_*x*_. A significant current drop (−88.23% compared to its original value) can be observed from the HF‐etched Nb_2_CT_*x*_ after 5th cyclic voltammetry (CV) scan as shown in Figure 4f and Figure S10 in the Supporting Information, while only a minor decrease (−0.925% compared to the initial current density) is registered from our E‐etched Nb_2_CT_*x*_. We suspected the tendency to be oxidized of the HF‐etched Nb_2_CT_*x*_ and the subsequent formation of NbO_*x*_ species onto MXene result in the significant decreases in anodic current after a few cycles. Such sharp comparison indicates an outstanding stability of E‐etched Nb_2_CT_*x*_ in an anodic potential window for various electrochemical sensing platforms. We further investigated the performances and feasibility of HF‐etched Nb_2_CT_*x*_ and E‐etched Nb_2_CT_*x*_‐based biosensor for OPs detection. As shown in Figure 4g, the nanoplatform based on E‐etched Nb_2_CT_*x*_ has much higher sensitivity when compared to the corresponding one from HF‐etched Nb_2_CT_*x*_. The primary reason is that the released HF from HF‐etched Nb_2_CT_*x*_ has an inhibition effect for the AChE, resulting in low electroactivity of choline and poor electron transfer ability. The developed E‐etched Nb_2_CT_*x*_‐based biosensing platform offers the capability of real‐time and on‐site detection. The properties of the GC/E‐etched Nb_2_CT_*x*_/GA/AChE for the OPs detection were characterized by chronoamperometric measurements. As shown in Figure S11 in the Supporting Information, the current response of the modified electrode in phosphate buffer solution with 40 µL (500 × 10^−6^ m) ATCh was recorded. After incubation in a series of different concentrations of pesticide solutions, the current signal was subsequently reduced, ascribed to the reduced catalytic activity caused by the irreversible binding between the OPs and the active sites of enzyme. The calibration plot is presented in Figure 4h, showing the inhibition rate (*I*%) increased linearly with the logarithm of phosmet concentration in the range of 200 × 10^−12^ to 1000 × 10^−9^ m for the proposed biosensor system with *r* ^2^ = 0.9501, fitted as *y = *0.1049*x* *+* 1.119. The LOD was calculated to be about 144 × 10^−12^ m (0.046 ng mL^−1^), which is much lower than those of the previously reported AChE‐based system (Table S2, Supporting Information) and the allowed maximum quantity (10 ppb) set by the U.S. Environmental Protection Agency.^[^ ^29^ ^]^ Thus, our proposed biosensing system possesses remarkable sensitivity for phosmet detection. Moreover, some possible interfering substances on vegetables and fruits were examined to evaluate the selectivity of the GC/E‐etched Nb_2_CT_*x*_/GA/AChE nanoplatform. These anions and cations have been tested and showed no obvious inhibition effect on the enzyme's activity of this biosystem (Figure 4i). Recovery measurement of phosmet‐spiked apple was then carried out and the recovery percentages of 98.05%, 105.57%, and 107.77% were obtained at phosmet concentrations of 1 × 10^−9^, 10 × 10^−9^, and 1000 × 10^−9^ m (Figure 4j and **Table** **1**), respectively. The results as above manifest a satisfactory repeatability and analytical performance in determining phosmet from real samples. The presented biosensor with desirable capability is indeed feasible and responsive for detecting low concentrations of real phosmet‐spiked samples.

**Figure 4:**
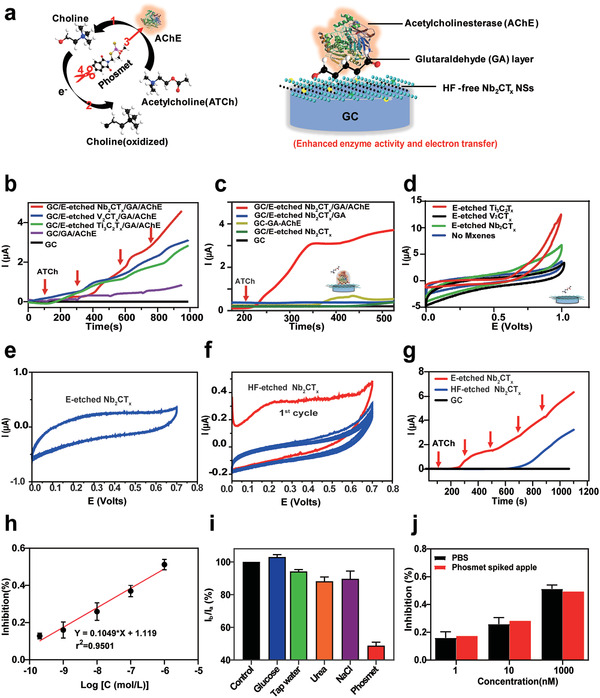
a) The enzyme inhibition effect for phosmet detection via HF‐free Nb_2_CT_*x*_/AChE based biosensor. b) Chronoamperometric measurements with successive injection of ATCh on different HF‐free MXenes modified biosensor. c) Chronoamperometric measurements with successive injection of ATCh on different modified layer of the proposed system. d) Electrocatalytic activity of the HF free MXene in presence of acetylcholine. The scan rate is 50 mV s^−1^. e) CV of CE/E‐etched Nb_2_CT_*x*_and f) CE/HF‐etched Nb_2_CT_*x*_showing the 1st to 5th CV scans run at a potential window from 0 to +0.7 V at a sweep rate of 10 mV s^−1^. g) Performance comparison of HF‐etched Nb_2_CT_*x*_and E‐etched prepared Nb_2_CT_*x*_based biosensor. h) Calibration plot showing the inhibition of biosensor versus phosmet concentration. i) The interference analysis of the anions and cations common in fruits. j) Recoveries with phosmet‐spiked apple for concentrations of 1 × 10^−9^, 10 × 10^−9^, and 1000 × 10^−9^ m. Arrows indicate the point of injection of ATCh (500 × 10^−6^ m), 0.1mPBS, pH = 6.5.

**Table 1:** Recovery measurement of phosmet‐spiked apple samples by GC/Fluorine‐free Nb_2_CT_*x*_‐AChE based electrochemical sensing platform

Sample^a)^	1	2	3	4
Phosmet spiked [nm]	0	1	10	1000
Phosmet determined [nm]	0.0050	0.9805	10.557	1077.7
Recovery [%]	–	98.05%	105.57%	107.77%

^a)^ nm indicates nmol of phosmet to 1L of 50 % ethanol aqueous solution.

In summary, we synthesized 2D fluorine‐free Nb_2_CT_*x*_ nanosheets via a facile strategy of E‐etching exfoliation. The Nb_2_CT_*x*_ obtained through this rapid and safe synthesis method has good chemical stability, low cytotoxicity, high porosity, and large surface area and enhanced conductivity. More importantly, the E‐etched Nb_2_CT_*x*_ with an environmentally friendly surface is well dispersed in aqueous solution to further stabilize the enzyme activity, thereby satisfying the requirements in potential biosensing applications. Consequently, we engineered the E‐etched Nb_2_CT_*x*_ nanosheets/AChE‐based nanoplatform as an electrochemical biosensor for OPs detection that exhibits much enhanced enzyme activity and electron transfer ability in comparison to conventional HF‐etched Nb_2_CT_*x*_. Benefiting from these merits, the fabricated biosystem shows the advantages of ultrahigh sensitivity, selectivity, outstanding anti‐interference ability, admirable recovery percentages in real sample analysis and good linearity with an LOD as low as 0.046 ng mL^−1^, which far exceeds the standard set by the U.S. Environmental Protection Agency. This work is the first time to explore biosensing application using fluoride‐free MXene, opens a new avenue to conceive other biomedical applications, such as NIR‐II photothermal therapy and photoacoustic imaging.

## Experimental Section

##### Materials and Methods

Potassium hexacyanoferrate (III), potassium hexacyanoferrate (II) trihydrate, 25% GA, acetylcholine iodide (ATCh), acetylcholinesterase (AChE) from electric eel, nitric acid, and phosphate buffer saline (PBS) were purchased from Sigma‐Aldrich. Phosmet was obtained from J&K Scientific (Hong Kong) Ltd. Nb_2_AlC (>99%) was obtained from Laizhou Kai Ceramic material Co., Ltd. Carbon fiber cloths (CFCs) were purchased from CeTech (W0S1002) with a thickness of 0.33 mm. The electrochemical characterizations were performed by using Solartron Electrochemical workstation with standard three electrode system in 1 m PBS (aq). All these chemicals were used as received without further purification.

##### Surface Modification of CFCs

The CFCs were treated with nitric acid surface modification and then were washed under sonication in acetone and ethanol to remove their surface organic grease. Afterward, the CFCs were immersed in concentrated nitric acid (63 wt%, Sigma) with refluxing at 125 °C for 3 h. Afterward, the CFCs were neutralized with 1 m NaOH(aq).

##### Fabrication of E‐Etched MXenes

All E‐etched MXenes studied in this work were synthesized by E‐etching method in a standard three electrode configuration. The 3D composite electrode was fabricated by mixing MAX phase powder (Ti_3_AlC_2_,V_2_AlC, and Nb_2_AlC, >99%, Laizhou Kai Ceramic material Co., Ltd) and conductive carbon black (CB) at a mass ratio of 95: 5 was directly dispersed in 1 mL poly(vinyl alcohol) (1 wt%) as a binder and uniformly dropped‐cast onto the CFC substrate. By the anodization to the Al layer under 1 V for 4 h at 0.5 m HCl electrolyte at 50 °C, the Al layer was selectively removed. The TIMCAL SUPER P Li CB was purchased from TangFeng Tech. Inc. with a diameter of 40 nm and a surface area of 62 m^2^ g^−1^. After that, 10 mg mixture was grinded and sonicated for 30 min to ensure that the powder was mixed properly. 3D electrode thermoassisted E‐etching was employed for selective removal of the Al layer from the Nb_2_AlC precursor, as proposed in the following equation

(1) $$\begin{array}{ccc} Nb_{2}\mathrm{AlC}+yCl^{-}+\left(2x+z\right)H_{2}O & \to & Nb_{2}C{\left(\mathrm{OH}\right)}_{2x}Cl_{y}O_{z}+Al^{3+}+\left(x+z\right)H_{2}\\ & & ↑+\left(y+3\right)e^{-} \end{array}$$

##### Purification of E‐Etched Mxenes

The freshly etched MXenes were collected from the CFC by rinsing the composite electrode with ethanol and sonication for ≈20 s. Then, the product mixture was first purified by sonication at room temperature for 30 min and centrifuge at 2000 rpm for 5 min. The supernatant containing MXene sheets were collected by decantation and centrifuge at 9000 rpm for 10 min. The sheets were further purified with ethanol and sonication for several times to remove unetched MAX phase material and CB. Finally, ≈5 mg MXene sheets were collected.

##### Fabrication of HF‐Etched Mxene

Nb_2_AlC powders were immersed in 50% aqueous hydrofluoric solution for 90 h at 55 °C. The resulting MXene suspension was repeatedly washed with deionized water (DI) water and centrifuged at 3500 rpm until the pH reached ≈ 6. The final portion of MXene suspension was washed with 50 mL of DI water.

##### Electrochemical Measurement

The electrochemical characterizations were evaluated by using Solartron Electrochemical workstation with standard three electrode system in 1 m PBS (aq). Platinum (Pt), Ag/AgCl, and GC electrodes (diameters of 3 mm) were obtained from Jingke Instruments (Shanghai, China). CV experiment was performed at a scan at rate of 100 mV s^−1^.

##### Modification of Biosensor System

AChE was dissolved in 0.1 m PBS. 10 µL (1 mg mL^−1^) E‐etched MXenes were dropped on the glassy carbon (GC) electrode surface and stayed dry at room temperature. 25% GA layer was casted onto the GC‐E‐etched MXene surface. 4 µL AChE were then coated onto the surface of GC‐E‐etched MXene‐GA. Glutaraldehyde was used and served as a crosslinker between the protein groups in AChE and E‐etched MXene to hold the enzymes in place as well as provide structural stability to the biosensor system. Every coated step was dried for 30 min. The degree of inhibition can be measured based on the change in choline signal response before and after phosmet inhibition. The inhibition percentage (*I*%) was calculated to evaluate the extent of inhibition before *(I*_b_) and after incubating (*I*_a_) with phosmet (Equation (2)) to analyze the inhibition activity of phosmet on AChE. A higher *I*% indicates greater inhibition of enzymes, which correlates to better performance of the biosensor.

(2) $$\begin{array}{c} I\%=\frac{I_{b}-I_{a}}{I_{b}}\times 100\% \end{array}$$

##### The Calculation of Recovery Rate of Phosmet‐Spiked onto Apples

Fresh apples were purchased from local supermarket, 5 mL of phosmet stock solutions with different concentrations were sprayed on organic apples. After the phosmet was exposed for 12 h, these apple samples were washed with 10 mL of ethanol for three times, and the eluents were collected. Then, the GC‐E‐etched Nb_2_CT_*x*_‐GA‐AChE biosensing platform was used to calculate the percentage of inhibition. Finally, the recovery rate of phosmet‐spiked onto apples was obtained by calculating the ratio of the detected phosmet amount to the theoretically calculated phosmet one.

##### Characterizations

Powder XRD patterns of various MXenes were recorded using a Rigaku smart lab 9 kW (Rigaku, Japan) with Cu *K*_*α*_ radiation (*λ* = 0.15 406 nm). The morphology and size of Nb_2_AlC and MXenes were characterized by using JEOL Model JSM‐6490 SEM equipped with an Oxford Instrument energy dispersive X‐ray spectrometry system, separated at 200 kV. XPS analysis was conducted in the system of a Sengyang SKL‐12 electron spectrometer equipped with a VG CLAM 4MCD electron energy analyzer. Al *K*_*α*_ source (1253.6 eV) operated at an accelerating voltage of 10 kV and emission current of 15 mA. The Raman spectra of the samples were obtained from a Witec Confocal Raman system equipped with an excitation source of continuous wave 532 nm diode laser.

## Conflict of Interest

The authors declare no conflict of interest.

## Supporting information

Supporting Information
