# Supplementary material for: Fluoride‐Free 2D Niobium Carbide MXenes as Stable and Biocompatible Nanoplatforms for Electrochemical Biosensors with Ultrahigh Sensitivity
Source: Adv Sci (Weinh). 2020 Nov 9;7(24):2001546. doi: 10.1002/advs.202001546 (PMC7739949; doi:10.1002/advs.202001546)
Supplement: Supplementary file 1 — Supporting Information [file ADVS-7-2001546-s001.pdf]

© 2020 Wiley-VCH GmbH

ADVANCED SCIENCE
Open Access

Supporting Information

for *Adv. Sci.*, DOI: 10.1002/advs.202001546

Fluoride-Free 2D Niobium Carbide MXenes as Stable and Biocompatible Nanoplatforms for Electrochemical Biosensors with Ultrahigh Sensitivity

*Menglin Song, Sin-Yi Pang, Feng Guo, Man-Chung Wong, and Jianhua Hao**

Copyright © 2020 Wiley-VCH GmbH

Supporting information

Fluoride-free 2D niobium carbide MXene as stable and biocompatible nanoplatforms for electrochemical biosensor with ultrahigh sensitivity

*Menglin Song, Sin-Yi Pang, Feng Guo, Man-Chung Wong, Jianhua Hao**

Table S1. Comparisons of Etching hours of Nb_2CT_x MXenes synthesized by different methods.

Materials	Synthesis method	Time required	w/ or w/o F^-	Ref.
Nb_2CT_x	50% HF aqueous solution, 55 °C	48h	w/	[1]
Nb_2CT_x	50% HF aqueous solution, RT	72h	w/	[2]
Nb_2CT_x	50 % concentrated HF solution and stirred for 90h at RT. Complete etching: in a 55 °C HF solution for 48 h	90h or 48h	w/	[3]
Nb_2CT_x	E-etching with composite electrode, 1 V, 4 h, 50 °C in 0.5 M HCl electrolyte.	4h	w/o	This work

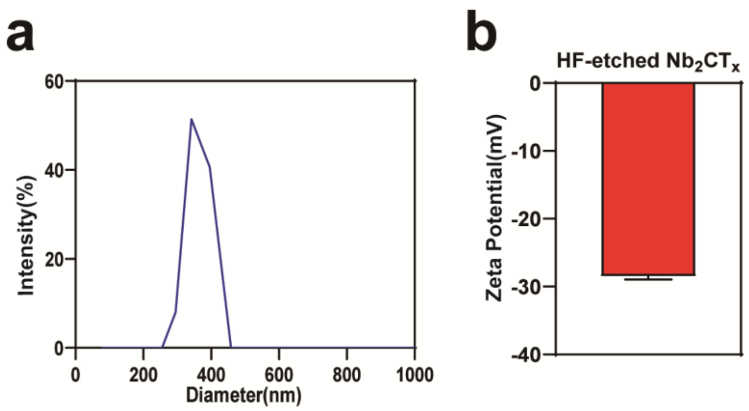

Figure S1. (a) Dynamic light scattering (DLS) size distribution profiles and (b) Zeta potential of HF-etched Nb_2CT_x in aqueous solution.

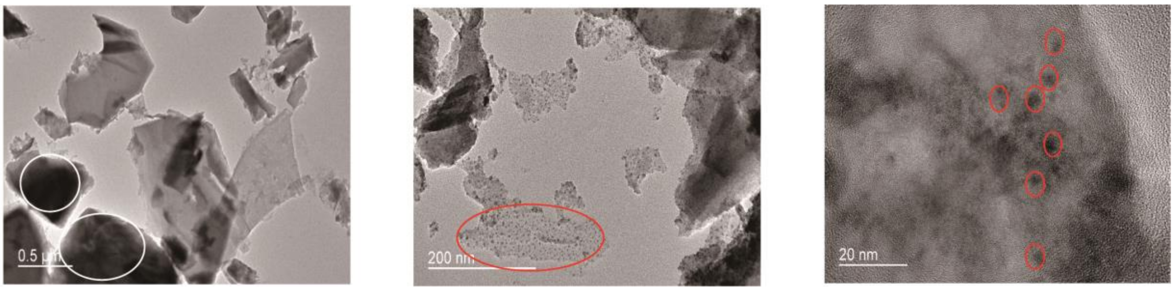

Figure S2. TEM images of HF-etched preparation of Nb_2CT_x MXenes with different magnifications. (white circles indicate the unsuccessful exfoliation of Al and the red circles indicate NbO_x nanoparticles formed by oxidation)

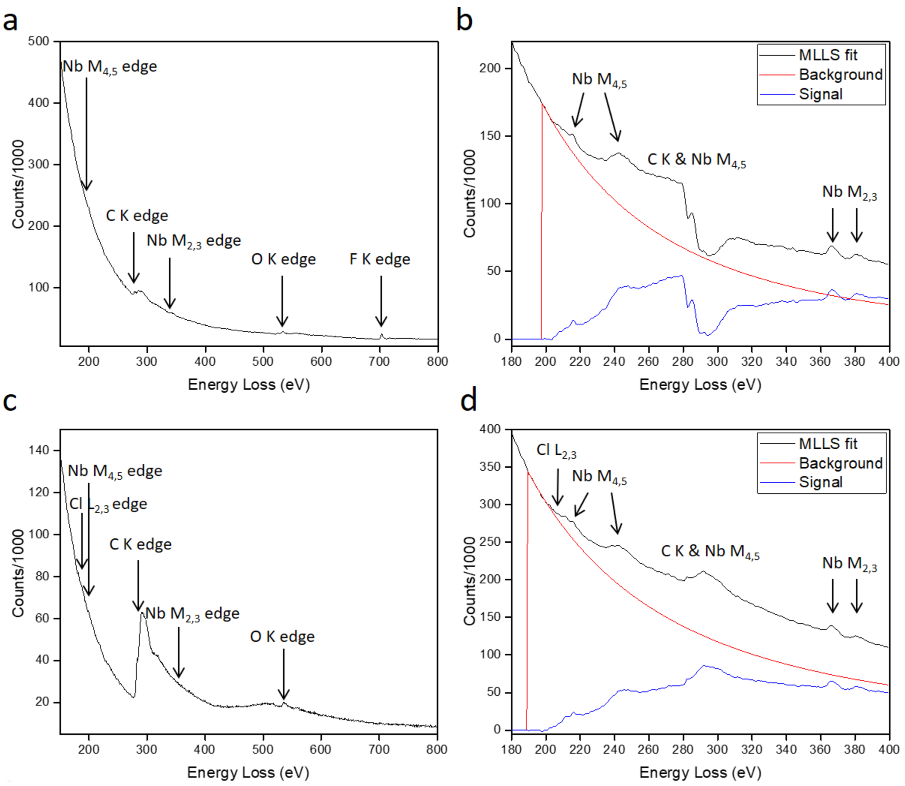

Figure S3. Corresponding electron energy loss spectrum (EELS) of HF-etching and E-etching Nb_2CT_x nanosheets for the Cl-$L_{2,3}$ edge at 200 eV, C-K edge at 284 eV, Nb-$M_{4,5}$ edge at 207 eV, Nb-$M_{2,3}$ edge at 371 eV, O-K edge at 532 eV and F-K edge at 685 eV.

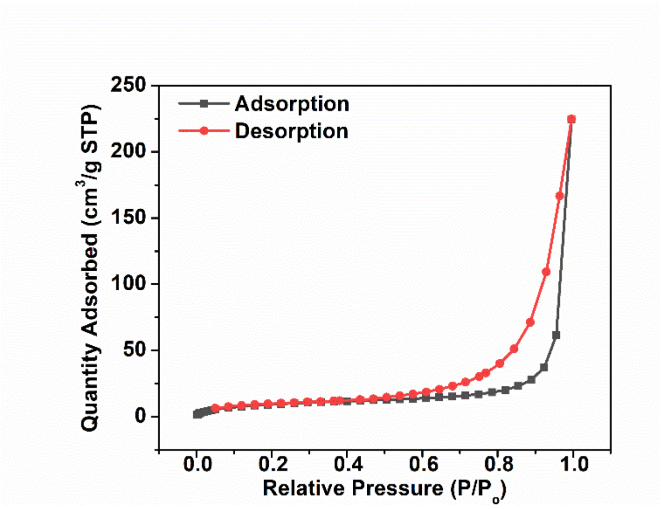

Figure S4. N_2 adsorption-desorption isotherm of E-etched Nb_2CT_x nanosheets.

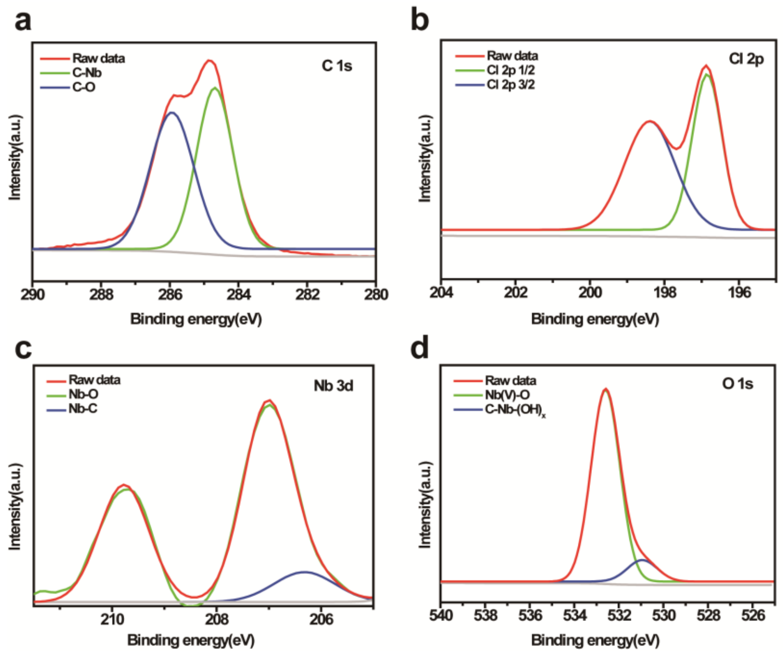

Figure S5. Core-level XPS spectra of C 1s (a), Cl 2p(b), Nb 3d (c), O1s (d) based on E-etched Nb_2CT_x.

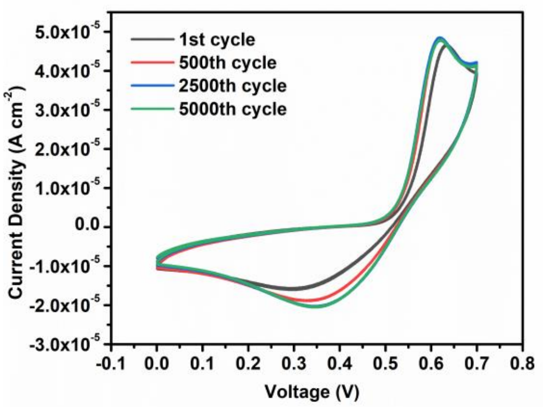

Figure S6. Cyclic voltammograms of GC/E-etched Nb_2CT_x/GA/AChE biosensor for 100 mV s^{-1} and number of scans up to 5000.

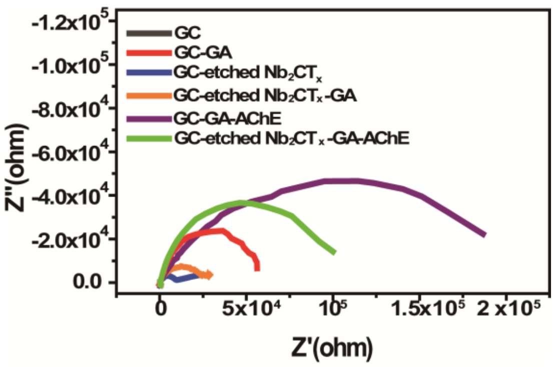

Figure S7. Nyquist plots of bare GC, GC- E-etched Nb_2CT_x MXenes, GC-E-etched Nb_2CT_x MXenes-GA, GC-GA-AChE and GC-E-etched Nb_2CT_x MXenes-GA-AChE in 0.1 M $K_3[Fe(CN)_6]/K_4[Fe(CN)_6]$ (1:1) containing 0.5 M KNO_3.

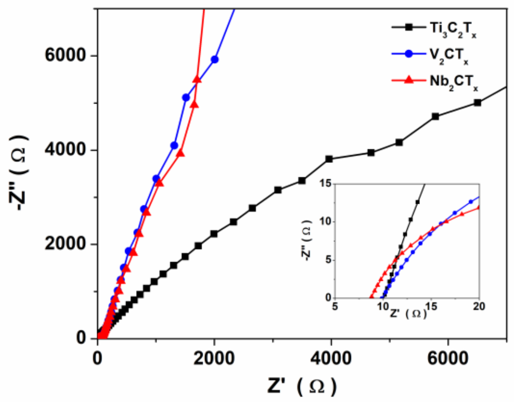

Figure S8. Electrochemical impedance spectroscope for HF-free $Ti_3C_2T_x$, V_2CT_x and Nb_2CT_x MXene. The inset shows the contact resistance of the electrode/electrolyte interface coupled with the corresponding MXene.

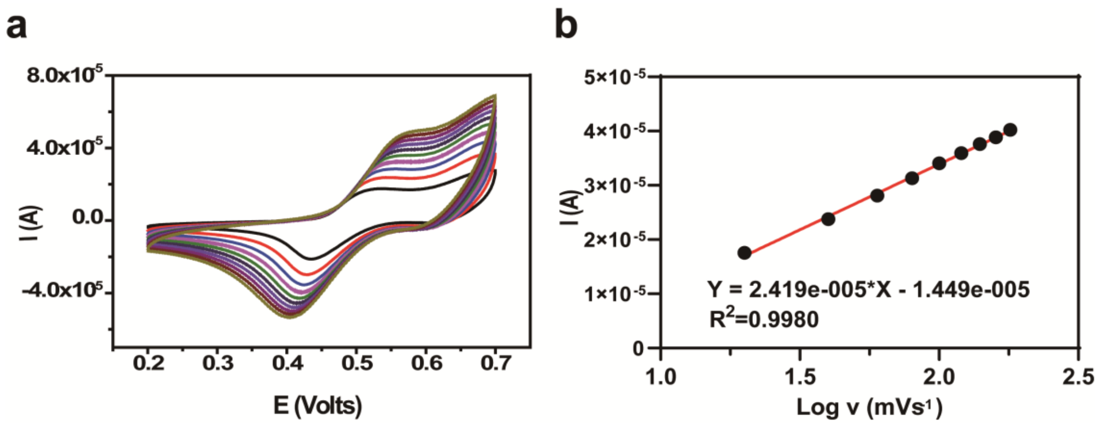

Figure S9. (a) Cyclic voltammograms of (10µL) 500 µM ATCh at different scan rates (20, 40, 60, 80, 100, 120 140 160 180 200 mV/s). (b) Linear relationship between the anodic peak currents with the scan rate. Conditions: 0.1 M PBS pH=6.5.

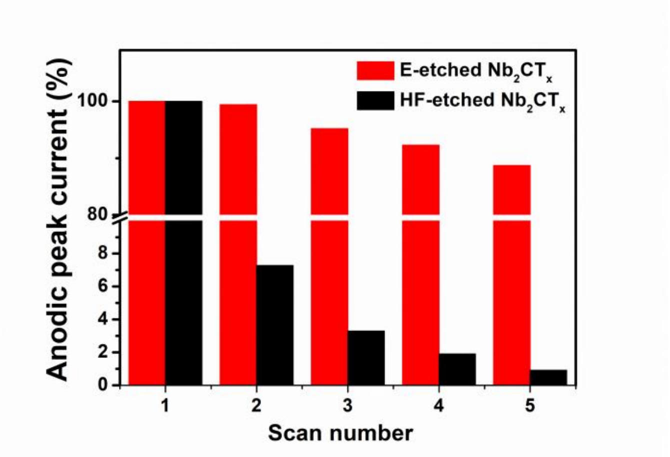

Figure S10. The bar chart of electrochemical stability of GCE/E-etched-Nb_2CT_x (red) and GCE/HF-etched-Nb_2CT_x (black) showing decrease in an anodic current read at +270 mV after five CV scans. (An anodic peak current at +270 mV corresponding to 100% for GCE/ E-etched-Nb_2CT_x was 2.21×10^{-7} A and for GCE/ HF-etched-Nb_2CT_x was 3.38×10^{-7}A). CVs were run at a potential window from 0 V to +0.7 V at a scan rate of 100 mV s^{-1}

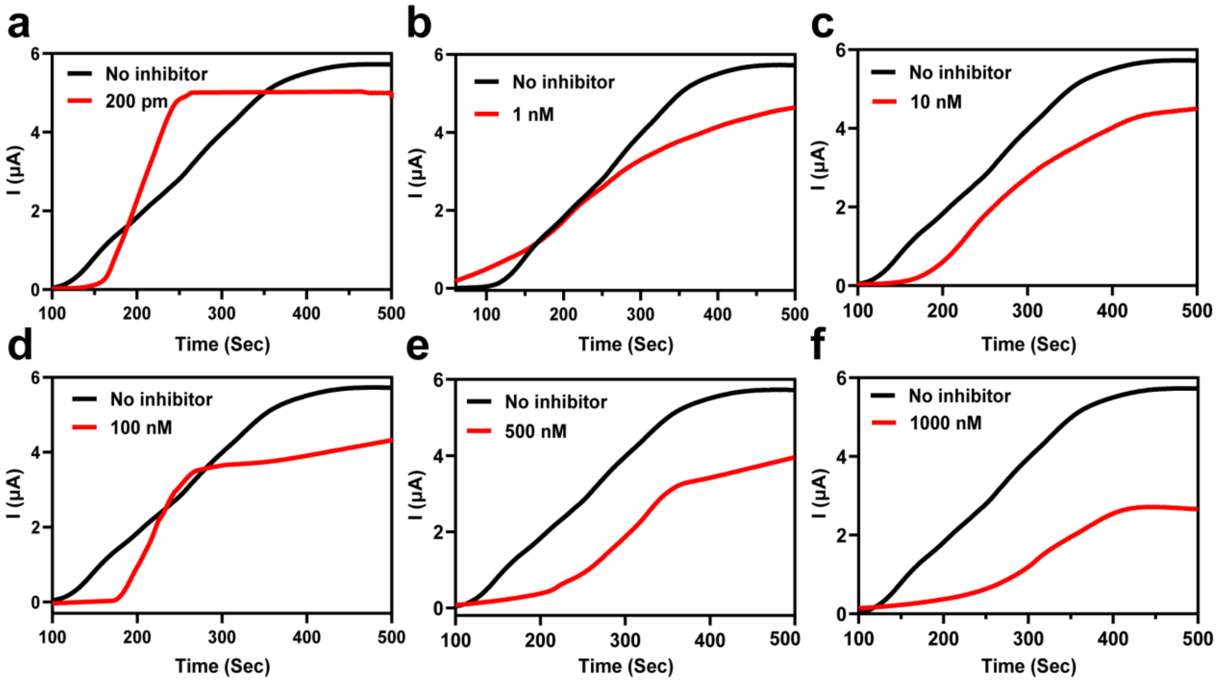

Figure S11. Chronoamperometric measurements at phosemet concentrations of a) 200 pM (b) 1 nM, (c) 1 nM, (d) 100 nM, and (e) 500 nM (f) 1000 nM in 0.01 M PBS and 250 µM ATCh. Black arrow indicates the point of injection of ATCh and blue arrow indicates the drop in current response observed after inhibition with phosmet.

Table S2. Comparisons of different systems for the detection of organophosphorus pesticides.

**Analytical system**	**Analytical method**	**LOD**	**Linear range**	**Ref.**
GC/WS_2/GA/AChE-BSA	Chronoamperometry	2.86 nM	1 - 1000 nM	[4]
SPE/CNT/CHO/AChE	Chronoamperometry	50 nM	1 – 200000 nM	[5]
GC/AChE-PAn-PPyMWCNTs	Chronoamperometry	3 nM	0.03-1.5 µM, 3-75µM	[6]
GC/HF-free Nb_2CT_x /GA/AChE	Chronoamperometry	1.44 nM	0.2 - 1000 nM	This work

References

[1] O. Mashtalir, M. R. Lukatskaya, M. Q. Zhao, M. W. Barsoum, Y. Gogotsi, *Adv. Mater.* **2015**, *27*, 3501-3506.

[2] H. Lin, S. Gao, C. Dai, Y. Chen, J. Shi, *J. Am. Chem. Soc.* **2017**, *139*, 16235-16247.

[3] M. Naguib, J. Halim, J. Lu, K. M. Cook, L. Hultman, Y. Gogotsi, M. W. Barsoum, *J. Am. Chem. Soc.* **2013**, *135*, 15966-15969.

[4] M. Z. M. Nasir, C. C. Mayorga-Martinez, Z. k. Sofer, M. Pumera, *ACS nano* **2017**, *11*, 5774-5784.

[5] Y. Lin, F. Lu, J. Wang, *Electroanalysis* **2004**, *16*, 145-149.

[6] D. Lu, J. Wang, L. Wang, D. Du, C. Timchalk, R. Barry, Y. Lin, *Adv. Funct. Mater.* **2011**, *21*, 4371-4378.
